# Effects of Diet and Phytogenic Inclusion on the Antioxidant Capacity of the Broiler Chicken Gut

**DOI:** 10.3390/ani11030739

**Published:** 2021-03-08

**Authors:** Eirini Griela, Vasileios Paraskeuas, Konstantinos C. Mountzouris

**Affiliations:** Laboratory of Nutritional Physiology and Feeding, Department of Animal Science, Agricultural University of Athens, Iera Odos 75, Athens 11855, Greece; eirinigkr21@gmail.com (E.G.); v.paraskeuas@gmail.com (V.P.)

**Keywords:** antioxidant response, Nrf2, phytogenics, diet type, broilers, gut health, animal nutrition

## Abstract

**Simple Summary:**

Triggering of poultry capacity to resist challenge stressors could be vital for animal performance and health. Diet may serve as a tool for modulating animal response to oxidative stress. Within the context of a balanced diet, certain feed additives of plant origin, such as phytogenics, may confer additional cytoprotective effects. As gut health is a prerequisite for animal performance, this work delved into advancing our knowledge on dietary and phytogenic effects on the capacity of the poultry gut to counteract oxidative stress. Study findings showed that a reduction in dietary energy and protein intake by 5% primed important antioxidant responses especially upon phytogenic addition. The new knowledge could assist in devising nutritional management strategies for counteracting oxidative stress.

**Abstract:**

The reduction in energy and protein dietary levels, whilst preserving the gut health of broilers, is warranted in modern poultry production. Phytogenic feed additives (PFAs) are purported to enhance performance and antioxidant capacity in broilers. However, few studies have assessed PFA effects on a molecular level related to antioxidant response. The aim of this study was to investigate the effects of administering two dietary types differing in energy and protein levels (L: 95% and H: 100% of hybrid optimal recommendations) supplemented with or without PFA (−, +) on gene expressions relevant for antioxidant response along the broiler gut. Interactions of diet type with PFA (i.e., treatments L−, L+, H−, H+) were determined for critical antioxidant and cyto-protective genes (i.e., nuclear factor erythroid 2-like 2 (Nrf2) pathway) and for the total antioxidant capacity (TAC) in the proximal gut. In particular, the overall antioxidant response along the broiler gut was increased upon reduced dietary energy and protein intake (diet type L) and consistently up-regulated by PFA addition. The study results provide a new mechanistic insight of diet and PFA functions with respect to the overall broiler gut antioxidant capacity.

## 1. Introduction

Gut health biomarkers associated with the regulation of the antioxidant response and inflammation currently attract a lot of scientific attention [1,2,3]. In particular, one of the most important regulators of antioxidant response and inflammation is the transcription factor nuclear factor erythroid 2-like 2 (*Nrf2*) [4]. Transcription factor *Nrf2* is a basic leucine zipper-containing transcription factor that is regulated by Kelch-like ECH-associated protein-1 (*Keap1*) and activates phase II/detoxifying enzymes and more than 100 genes through the antioxidant response element (ARE). These genes include NAD(P)H:quinone oxidoreductase 1 (*NQO1*), glutathione S-transferase (*GST*), heme oxigenase-1 (*HO-1*), glutathione peroxidase (*GSH-Px*), catalase (*CAT*), superoxide dismutase 1 (*SOD1*), glutamate cysteine ligase (*GCL*), glutathione-disulfide reductase (*GSR*) and the thioredoxin/peroxiredoxin system [5,6,7,8].

In particular, Keap1 binding with various inducers (e.g., phytogenic compounds) leads to transcription of many cyto-protective genes [9,10]. Briefly, CAT and SOD1 are antioxidant enzymes that directly react with radical species, whereas GPX and GSR regenerate oxidized antioxidants [11]. In addition, NQO1 engages a two-electron transfer to diminish quinones to hydroquinones preventing the production of free radical oxygen intermediates [12]. Moreover, GST catalyzes the conjugation of GSH with xenobiotics and protects cells against reactive oxygen metabolites [13]. On the other hand, PRDX1 is proven to be a functional enzyme adjusting cell growth, differentiation and apoptosis [14]. Finally, TXN operates along with PRDX1 as reductase in redox control, preserves proteins from oxidative aggregation and inactivation, supports the cells confront various environmental stresses (e.g., ROS, peroxynitrite, arsenate) and regulates programmed cell death via denitrosylation [15].

Research evidence highlights that activation of the *Nrf2*/ARE signaling pathway could be regarded as beneficial for effectively counteracting oxidative stress in animals and humans. In this respect, contemporary research tries to elucidate the role of dietary components for animal and human health and well-being [16]. In particular, while there is evidence that reduced energy and protein intake could be beneficial for adult humans [17,18], yet the role of energy and protein intake in the activation of the *Nfr2* pathway [19] is still limited. On the other hand, accumulating evidence demonstrates that inclusion of various phytogenic feed additives (PFAs) may regulate the *Nrf2*/ARE pathway in a manner perceived as beneficial for human and animal health [3,6,20,21,22].

The aim of this study was to generate new knowledge on the effects of dietary energy and protein levels with or without PFA addition on the modulation of the *Nrf2*/ARE signaling pathway in the broiler gut mucosa. For the purpose of the study, the expression of critical genes belonging to the *Nrf2*/ARE pathway was profiled along the chicken broiler gut. In addition to the gene expressions, the antioxidant capacity of the intestinal mucosa was assessed biochemically.

## 2. Materials and Methods

### 2.1. Animals and Experimental Treatments

For the purpose of the experiment, 540 one-day-old male Cobb 500 broilers vaccinated at hatch for Marek, Infectious Bronchitis and Newcastle Disease were obtained from a commercial hatchery. Birds were allocated to 4 experimental treatments for 6 weeks. Each treatment had 9 floor replicate cages of 15 broilers each. Each replicate was assigned to a clean floor cage (1 m^2^), and the birds were raised on rice hulls litter. The temperature program was set at 32 °C at week 1 and gradually reduced to 23 °C by week 6. Heat was provided with a heating lamp per cage. Except for day 1, an 18 h light to 6 h dark lighting program was applied during the experiment to ensure adequate access to feed and water.

A 2 × 2 factorial design was used with diet specifications and PFA addition as the main factors. A three-phase feeding program with starter (1 to 10 d), grower (11 to 22 d) and finisher (23 to 42 d) diets was followed. In particular, for each growth phase, two diet types () were formulated to meet 95% and 100% of optimal Cobb 500 metabolizable energy (ME) and protein (CP) specifications, stated as L and H, respectively. The PFA used contained a blend of compounds such as carvacrol, thymol, carvone, methyl salicylate and menthol (Digestarom^®^ Biomin Phytogenics GmbH, Stadtoldendorf, Germany). Diets were in mash form, based on maize and soybean meal and were supplemented with coccidiostat. Throughout the experiment, feed and water were available ad libitum.

The calculated chemical composition per kg of the basal diets (L vs. H) was as follows. For the starter diet: AMEn (11.97 vs. 12.60) MJ; crude protein (204.3 vs. 215.0) g; lysine (12.5 vs. 13.2) g; methionine + cysteine (9.4 vs. 9.9) g; threonine (8.2 vs. 8.6) g; calcium 9 g; available phosphorus 4.5 g. For the grower diet: AMEn (12.27 vs. 12.92) MJ; crude protein (185.3 vs. 195) g; lysine (11.3 vs. 11.9) g; methionine + cysteine (8.6 vs. 9.0) g; threonine (7.5 vs. 7.9) g; calcium 8.4 g; available phosphorus 4.2 g. For the finisher diet: AMEn (12.59 vs. 13.26) MJ; crude protein (175.8 vs. 185.0) g; lysine (10.0 vs. 10.5) g; methionine + cysteine (7.8 vs. 8.2) g; threonine (6.8 vs. 7.1) g; calcium 7.6 g; available phosphorus 3.8 g.

Depending on diet type (L and H) and PFA supplementation (0 and 150 mg/kg of diet), the four experimental treatments were: L− (95% of optimal ME and CP requirements with no PFA supplementation), L+ (95% of optimal ME and CP requirements with PFA supplementation), H− (100% of optimal ME and CP requirements with no PFA supplementation) and H+ (100% of optimal ME and CP requirements with PFA supplementation).

The experimental protocol was in compliance with the current European Union Directive on the protection of animals used for scientific purposes [23,24] and was approved by the relevant national authority (Department of Agriculture and Veterinary Policy, General Directorate of Agriculture, Economy, Veterinary and Fisheries). Birds were euthanized via electrical stunning prior to slaughter.

### 2.2. Broiler Growth Performance Responses

Broiler performance parameters such as body weight gain (BWG), feed intake (FI), and feed conversion ratio (FCR) were evaluated for the entire duration of the experiment (42 days) (Table 2).

### 2.3. Organ Sampling

At 42 d of age, 9 broilers per treatment were randomly selected and the duodenum, jejunum, ileum and ceca samples were excised carefully and immediately snap frozen in liquid nitrogen and subsequently stored at −80 °C for further analyses.

### 2.4. Molecular Analyses

#### 2.4.1. RNA Isolation and Reverse-Transcription PCR

Τhe central section of duodenum, jejunum, ileum and the whole ceca were exposed and the luminal digesta was ejected. Then, the segments without digesta were washed completely in 30 mL cold phosphate buffered saline (PBS)–ethylene diamine tetra-acetic acid (EDTA; 10 mmol/L) solution (pH = 7.2), and the mucosal epithelium was taken off with a micro-slide to a sterile Eppendorf type tube. Eventually, the total RNA from the duodenal, jejunal, ileal and caecal mucosa was obtained as reported by the manufacturer’s protocol from Macherey-Nagel GmbH & Co. KG, Duren, Germany, by handling NucleoZOL Reagent. RNA quantity and quality were ascertained by spectrophotometry with the use of NanoDrop-1000 by Thermo Fisher Scientific, Waltham, United Kingdom.

DNAse treatment was exercised due to the removal of contaminating genomic DNA from the RNA samples. Ten micrograms of RNA was diluted with 1 U of DNase I (M0303, New England Biolabs Inc, Ipswich, UK) and 10 μL of 10x DNAse buffer to a final volume of 100 µL upon the inclusion of DEPC water, for 15–20 min at 37 °C. Before the DNAse inactivation at 75 °C for 10 min, EDTA should be added to a final concentration of 5 mM to protect RNA from being degraded during enzyme inactivation. RNA integrity was examined by agarose gel electrophoresis

From each sample, 500 ng of total RNA was reverse transcribed to cDNA by PrimeScript RT Reagent Kit (Perfect Real Time, Takara Bio Inc., Shiga-Ken, Japan) according to the manufacturer’s recommendations. All cDNAs were afterwards stored at −20 °C.

#### 2.4.2. Quantitative Real-Time PCR

The following *Gallus gallus* genes were examined: nuclear factor erythroid 2-like 2 (*Nrf2*), kelch-like ECH associated protein 1 (*Keap1*), catalase (*CAT*), superoxide dismutase 1 (*SOD1*), xanthine oxidoreductase (*XOR*), glutathione peroxidase 2, 7 (*GPX2, GPX7*), heme oxygenase 1 (*HMOX1*), NAD(P)H quinone dehydrogenase 1 (*NQO1*), glutathione S-transferase alpha 2 (*GSTA2*), glutathione-disulfide reductase (*GSR*), peroxiredoxin-1 (*PRDX1*), thioredoxin (*TXN*), glyceraldehyde 3-phosphate dehydrogenase (*GAPDH*) and actin beta (*ACTB*). Suitable primers were designed using the GenBank sequences deposited on the National Center for Biotechnology Information and US National Library of Medicine (NCBI) shown in Table 1. Primers were checked using the PRIMER BLAST algorithm for *Gallus gallus* mRNA databases to ensure that there was a unique amplicon.

Real-time PCR was accomplished in 96-well microplates with a SaCycler-96 Real-Time PCR System (Sacace Biotechnologies s.r.l.,Como, Italy) and FastGene IC Green 2x qPCR universal mix (Nippon Genetics, Tokyo, Japan). Every reaction included 12.5 ng RNA equivalents along with 200 nmol/L of forward and reverse primers for each gene. The reactions were incubated at 95 °C for 3 min, accompanied by 40 cycles of 95 °C for 5 s, 59.5 to 62 °C (depending on the target gene) for 20 s, 72 °C for 33 s. This was tailed by a melt curve analysis to check the reaction specificity. Each sample was determined in duplicates. Relative expression ratios of target genes were calculated according to [25] adapted for the multi-reference genes normalization procedure according to [26] using GAPDH and ACTB as reference genes.

### 2.5. Biochemical Analyses

Total Antioxidant Capacity of Intestinal Mucosa

Total antioxidant capacity (TAC) was determined using the oxygen radical absorbance (ORAC) assay [27] to evaluate the hydrophilic antioxidants [28]. Appropriately diluted mucosal samples from duodenum, jejunum, ileum and caecum in phosphate-buffered saline (PBS) were used, and the ability to delay the decay of phycoerythrin fluorescence under the presence of 2,2′-azobis (2-methylpropionamidine) dihydrochloride (APPH) used as oxidant was compared with that of trolox (6-hydroxy-2,5,7,8 tetramethylchroman-2-carboxylic acid) used as an anti-oxidant standard. Data were expressed as concentration of trolox equivalents (TE) (mmol/L of serum).

### 2.6. Statistical Analysis

Experimental data were tested for normality using the Kolmogorov–Smirnov test and found to be normally distributed. Data were analyzed with the general linear model (GLM)–general factorial ANOVA procedure using diet type (L, H) and PFA addition (NO and YES) as fixed factors. Statistically significant effects were further analyzed, and means were compared using Tukey’s honestly significant difference multiple comparison procedure. Statistical significance was determined at *p* ≤ 0.05. All statistical analyses were performed using the SPSS for Windows Statistical Package Program (SPSS 17.0, Inc., Chicago, IL, USA).

## 3. Results

### 3.1. Growth Performance Responses

Significant interactions between diet type and PFA were found for BWG (*P*_D×P_ = 0.001) and FCR (*P*_D×P_ = 0.024). In particular, broilers of treatments H- and H+ had higher BWG and lower FCR values compared to treatments L− and L+, while broilers of treatment L+ had better BWG and FCR compared to treatment L−. Moreover, broilers fed diet type H showed higher (*P*_D_ < 0.001) BWG, FI (*P*_D_ = 0.046) and lower FCR (*P*_D_ < 0.001) compared to broilers fed diet type L. In addition, PFA inclusion significantly increased BWG (*P*_P_ = 0.002) and improved FCR *(P_P_* = 0.043) for the whole experiment (Table 2).

### 3.2. Profile of Selected Gene Expression along the Intestine

#### 3.2.1. Duodenum

**Figure 1:**
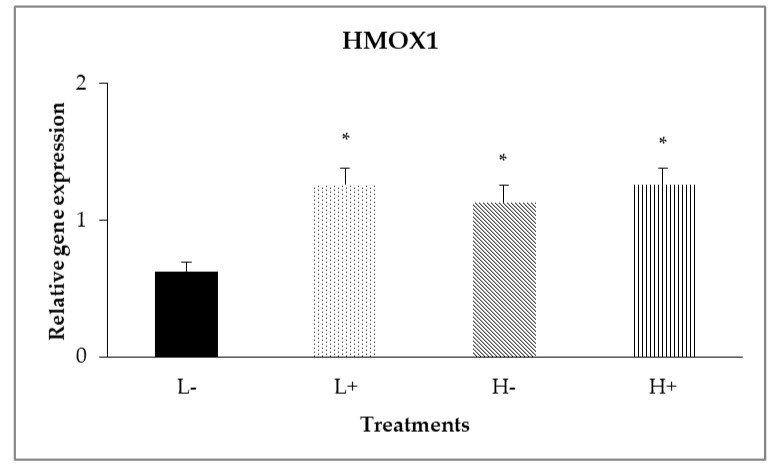
Interaction effects of diet type and phytogenic feed additive (PFA) supplementation on relative gene expression of *HMOX1* in the duodenal mucosa of 42-day-old broilers. Columns indicate treatments means + SD and the asterisks denotes statistical difference (*P*_D×P_ = 0.017).

In the duodenal mucosa, significant interaction (*P*_D×P_ = 0.017) was shown (Figure 1) between diet type and PFA inclusion for *HMOX1* gene expression levels, with broilers of treatment (L−) having lower relative gene expression compared to the other treatments.

Moreover, as shown in Table 3, diet type significantly affected (*P* < 0.05) relative gene expression of *GPX2* (*P*_D_ = 0.043) and *HMOX1* (*P*_D_ = 0.017) with broilers fed diet type L showing higher expression compared to broilers on diet type H. In addition, diet type affected the relative gene expression of *TXN* (*P*_D_ = 0.017) with broilers fed diet type H showing higher expression levels compared to broilers fed diet type L. In addition, PFA inclusion significantly (*P* < 0.05) up-regulated relative expression levels of *Keap1* (*P*_P_ = 0.001), *CAT* (*P*_P_ = 0.035), *SOD1* (*P*_p_ = 0.019), *HMOX1* (*P*_p_ = 0.001), NQO1 (*P*_p_ = 0.001), GSR (*P*_p_ = 0.041), *PRDX1* (*P*_p_ = 0.019) and *TXN* (*P*_p_ = 0.035). Gene expression of *Nrf2*, *XOR*, *GPX7* and *GST* was not significantly affected (*P* > 0.05) by PFA inclusion of diet type

#### 3.2.2. Jejunum

In the jejunal mucosa, diet type significantly affected *GPX2* (*P*_D_ = 0.005) and *PRDX1* (*P*_D_ = 0.002), with broilers fed diet type L showing higher expression levels compared to broilers fed diet type H, as presented in Table 4. On the other hand, relative gene expression of *Nrf2*, *Keap1*, *CAT*, *SOD1*, *XOR*, *GPX7*, *HMOX1*, *NQO1*, *GST*, *GSR* and *TXN* was not significantly affected (*P* > 0.05) neither by diet type nor PFA inclusion

#### 3.2.3. Ileum

**Figure 2:**
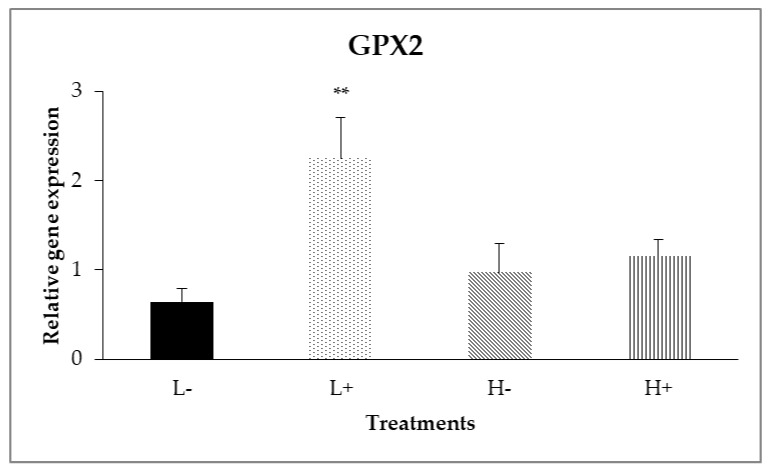
Interaction effects of diet type and phytogenic feed additive (PFA) supplementation on relative gene expression of *GPX2* in ileal mucosa of 42-day-old broilers. Columns indicate treatments means + SD, and the asterisk(s) denotes statistical difference (*P*_D×P_ = 0.007).

**Figure 3:**
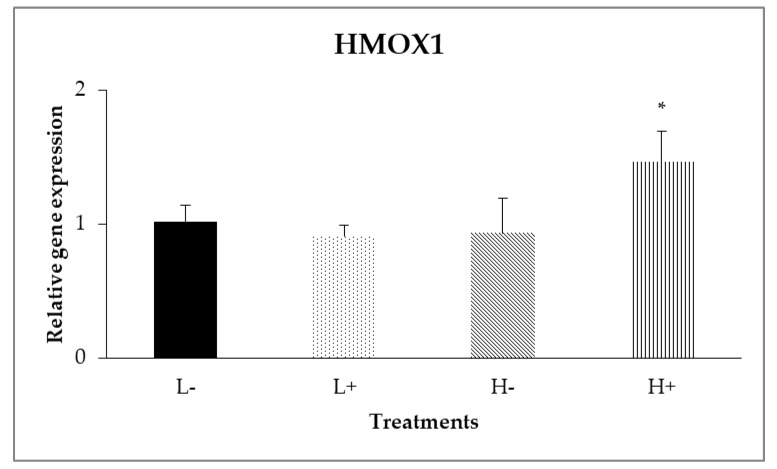
Interaction effects of diet type and phytogenic feed additive (PFA) supplementation on relative gene expression of *HMOX1* in ileal mucosa of 42-day-old broilers. Columns indicate treatments means + SD, and the asterisk denotes statistical difference (*P*_D×P_ = 0.024).

In the ileal mucosa, significant interactions between diet type and PFA inclusion were noted for *GPX2* (*P*_D×P_ = 0.007) and *HMOX1* (*P*_D×P_ = 0.024) as shown in Figure 2 and Figure 3. In particular, the highest relative expression level of *GPX2* was found on the L+ treatment, whereas on *HMOX1*, treatment H+ had the higher gene expression level compared to the other treatments.

In addition, diet type significantly affected (*P*_D_ = 0.006) the expression of *CAT*, with broilers fed diet type L showing higher expression levels compared to broilers fed diet type H. Moreover, as displayed in Table 5, PFA inclusion, significantly up-regulated *(P*_P_ < 0.001) the relative gene expression of *GPX2*. Finally, relative gene expression of *Nrf2, Keap1*, *SOD1*, *XOR*, *GPX7*, *NQO1*, *GST*, *GSR*, *PRDX1* and *TXN* was not significantly affected (*P* > 0.05) neither by diet type nor PFA inclusion

#### 3.2.4. Ceca

In the cecal mucosa, as shown in Table 6, diet type significantly affected (*P* < 0.05) the gene expression levels of *Keap1* (*P*_D_ = 0.014), *GPX2* (*P*_D_ = 0.003), *GPX7* (*P*_D_ = 0.032) and *PRDX1* (*P*_D_ = 0.006), with broilers fed diet type L showing higher expression levels compared to broilers fed diet type H. Moreover, PFA inclusion significantly up-regulated (*P*_P_ = 0.041) relative gene expression level of *GST*. However, relative gene expression of *Nrf2*, *Keap1*, *CAT*, *SOD1*, *XOR*, *HMOX1*, *NQO1*, *GSR* and *TXN* was not significantly affected (*P* > 0.05) neither by diet type nor PFA inclusion

### 3.3. Total Antioxidant Capacity (TAC) along the Intestine

**Figure 4:**
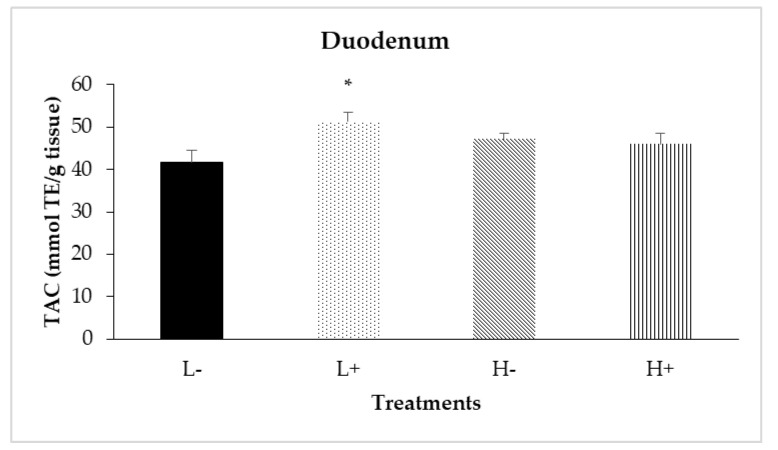
Interaction effects of diet type and phytogenic feed additive (PFA) supplementation on total antioxidant capacity (TAC) of duodenal mucosa of 42-day-old broilers. Columns indicate treatments means + SD, and the asterisk denotes statistical difference (*P*_D×P_ = 0.024).

**Figure 5:**
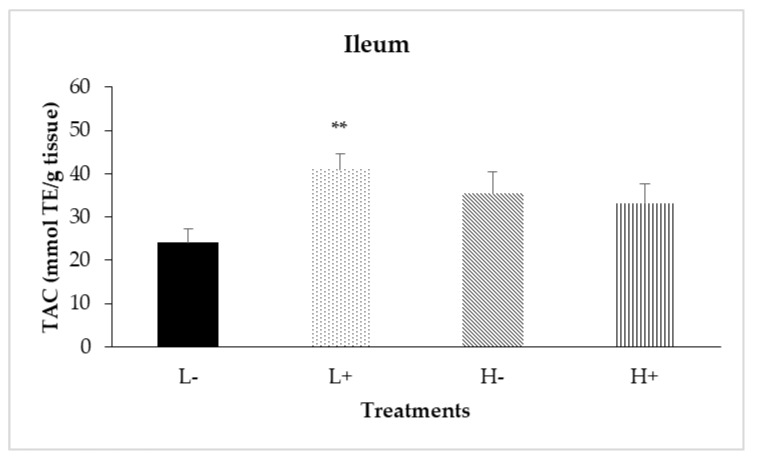
Interaction effects of diet type and phytogenic feed additive (PFA) supplementation on total antioxidant capacity of ileal mucosa of 42-day-old broilers. Columns indicate treatments means + SD, and the asterisk(s) denotes statistical difference (*P*_D×P_ = 0.007).

Significant interactions between diet type and PFA inclusion for TAC were noted in the duodenal (*P*_D×P_ = 0.024) and ileal (*P*_D×P_ = 0.007) mucosa as shown in Figure 4 and Figure 5, respectively. In particular, treatment L+ had higher TAC compared to the other treatments. In addition, diet type significantly affected TAC in jejunal mucosa (*P*_D_ = 0.001) with broilers fed diet type H having higher TAC compared to broilers fed diet type L.

Finally, as presented in Table 7, PFA inclusion significantly increased TAC in the jejunal (*P*_P_ < 0.001), ileal (*P*_P_ = 0.033) and cecal (*P*_P_ = 0.032) mucosa.

## 4. Discussion

A deeper understanding of the effects of dietary energy and protein levels on broiler gut function and health is still warranted. In this study, overall BWG and FCR were better in chickens receiving diet type H compared to diet type L (Table 2). These results were in line with previous studies regarding effects on performance and relevant biological responses [29,30,31,32]. However, the topic of energy and protein reduction on metabolic pathways related to cyto-protection via the Nrf2/ARE pathway is still scarce in broilers. On the other hand, there are indications for beneficial effects of dietary energy and protein reductions on the *Nrf2*/ARE pathway for adult humans [10,11].

In this study, overall body weight gain and FCR were improved in chickens receiving PFA, especially in the case of diet type L, whereas chickens in treatment L+ were better compared to L− (Table 2). Similar results have been previously reported [1,29,31]. Phytogenic compounds beyond their benefits for growth performance, nutrient digestibility and meat antioxidant capacity in broilers [1,29,31,33] are currently gaining attention for their functional role on critical elements of gut barrier integrity and inflammation [34,35,36]. Interestingly, emerging evidence reveals that PFA may modulate beneficially the *Nrf2*/ARE pathway in the broiler gut [3,37].

Therefore, the present study aimed to generate new knowledge regarding the effects of dietary energy and protein levels in conjunction or not with PFA supplementation on the stimulation of antioxidant and cyto-protective enzymes, via the activation of *Nrf2*/ARE pathway. For this reason, this study has employed a powerful analytical palette of gene coding for cytoprotective factors and enzymes in order to profile diet and PFA effects along the broiler gut. The genes and factors studied include a number of phase-2 proteins and antioxidant gene components of the Nrf2 signaling pathway (i.e., *Nrf2*, *Keap1*, *CAT*, *SOD1*, *XOR*, *GPX2*, *GPX7*, *HMOX1*, *NQO1*, *GST*, *GSR*, *PRDX1* and *TXN).*

In this study, the overall feed intake did not differ significantly between the experimental treatments. Therefore, according to diet specifications, birds on the low specification diet (i.e., treatments L and L+) had indeed lower overall ME and CP intake by approximately 5%, compared to birds on the high dietary specification (i.e., treatments H and H+).

Data analysis revealed interactions between diet type and PFA inclusion for *HMOX1* and *GPX2.* In particular, the low diet specs in combination with PFA supplementation up-regulated the *GPX2* gene in the ileal intestinal segment. Meanwhile, *HMOX1* in duodenum had shown a significant up-regulation in all treatments compared to L−. However, in ileum relative gene expression of *HMOX1* was increased only in H+ treatment. Regarding the interactions between diet and PFA, the biological significance of a single gene changes (*HMOX1*) by its own is questionable and could be considered with the findings about *GPX2* gene and TAC results in ileum (Table 5) that indicate PFA role in improving antioxidant capacity. Concerning the segment dependence for gene responses, PFA constituents had been shown to be mainly absorbed in the proximal gut (e.g., stomach and duodenum) [38]. As it was observed in this study, the examined genes that were up-regulated in duodenum upon PFA addition were above 60% (8/13 genes).

Moreover, reduced energy intake has been shown to intensify the repairment of DNA systems, advocate the elimination of damaged proteins and oxidized lipids and increase antioxidative defense mechanisms in humans [16], rats and monkeys [39,40]. The results of the present study support the modulatory effects of lower ME and CP intake towards an improved broiler anti-oxidative status. In particular, the low diet specs up-regulated the expression of genes relevant for cyto-protection (i.e., *Keap1*, *GPX2*, *GPX7* and *PRDX1*) mainly at the cecal level. In addition, benefits of reduced ME and CP diet specs for other gut health biomarkers (i.e., TLR, tight junctions) have also been shown previously in broiler ceca [36].

In this study, PFA supplementation resulted in *Keap1* up-regulation in the duodenum. This could be considered relevant for overall gut inflammation management since Keap1, besides its active participation in the Nrf2 pathway [41], also inhibits *NF-κΒ* via binding to its activator protein Ikkb [42]. Up-regulation of *Keap1* and down-regulation of *NF-κΒ* have recently been shown in the case of a PFA dose response study in broilers [3].

The PFA inclusion in this study up-regulated *CAT* and *SOD1* expressions in the duodenal mucosa. Increased *CAT* and *SOD1* activity have been shown in broiler blood following PFA addition [43], whereas [44] observed a significant up-regulation in these two enzymes with oregano essential oil (carvacrol) addition in porcine small intestinal epithelial cells. Furthermore, a significant up-regulation of *SOD1* expression in duodenal, jejunal and cecal mucosa has been shown in a PFA dose response study in broilers [3].

Irrespective of diet type, the administration of PFA up-regulated *NQO1* in the duodenal mucosa. In another study, an up-regulated *NQO1* expression in the duodenum was also shown following PFA supplementation [3]. NQO1, as mentioned earlier, catalyzes the two-electron mediated reduction of quinones to hydroquinones, which is commonly proposed as a mechanism of detoxification [12].

Furthermore, in this study, PFA inclusion resulted in increased *GSR, PRDX1* and *TXN* expressions in duodenal mucosa. Although there are no other relevant studies to compare directly, *PRDX1* up-regulation has also been reported in the case of other gut function modulating additives such as mannan-oligosaccharides in young broilers chickens [45].

From all the above, it appears that there are differences regarding the intensity and intestinal site specificity of PFA modulation of the Nrf2 pathway components, between various studies [3,36,37]. It is possible that phytogenic composition and inclusion level, as well as the absorption and metabolism kinetics of phytogenic active components within the birds, could possibly account for the differences between studies. However, the required knowledge on these topics is still rather limited.

The effects of PFA on the broiler intestine at a molecular level have been recently shown to correlate with increased intestinal mucosa TAC [3]. Similarly, in this study, PFA inclusion resulted in increased TAC in the jejunum, ileum and ceca. Overall, both studies above provide evidence for PFA cytoprotective and anti-oxidative potential at the broiler intestine.

## 5. Conclusions

In conclusion, this study has confirmed our previous findings on performance [1,31] and provided new knowledge for the effects of diet ME and CP specs on host antioxidant response. We found that a more intense priming of host antioxidant response was seen in birds fed the diets with ME and CP reduced by 5% of the recommended optimal dietary specifications for the broiler genetic line used. Moreover, beyond the known PFA benefits for performance [1,31], the study results have highlighted the PFA potential for host antioxidant protection, detoxification and inflammation management at intestinal level. Interestingly, it was shown that when PFA was used in conjunction with the low specifications diet, the cyto-protection potential at the intestine was maximized. From a human perspective, and given the higher than 60% homology between the *Gallus gallus* and the human genome [46], study findings could also be relevant for human gut health as contemporary dietary recommendations for reduced food intake and use of plant bioactive compounds increase in popularity.

## Figures and Tables

**Table 1:** Oligonucleotide primers used for gene expression of selected targets by quantitative real time PCR.

Target	Primer Sequence (5′-3′)	Annealing Temperature (°C)	PCR Product Size (bp)	GenBank (NCBI Reference Sequence)
*GAPDH*	F: ACTTTGGCATTGTGGAGGGT R: GGACGCTGGGATGATGTTCT	59.5	131	NM_204305.1
*ACTB*	F: CACAGATCATGTTTGAGACCTT R: CATCACAATACCAGTGGTACG	60	101	NM_205518.1
*Nrf2*	F: AGACGCTTTCTTCAGGGGTAG R: AAAAACTTCACGCCTTGCCC	60	285	NM_205117.1
*Keap1*	F: GGTTACGATGGGACGGATCA R: CACGTAGATCTTGCCCTGGT	62	135	XM_025145847.1
*CAT*	F: ACCAAGTACTGCAAGGCGAA R: TGAGGGTTCCTCTTCTGGCT	60	245	NM_001031215
*SOD1*	F: AGGGGGTCATCCACTTCC R: CCCATTTGTGTTGTCTCCAA	60	122	NM_205064.1
*XOR*	F:GTGTCGGTGTACAGGATACAGAC R:CCTTACTATGACAGCATCCAGTG	61	110	NM_205127.1
*GPX2*	F: GAGCCCAACTTCACCCTGTT R: CTTCAGGTAGGCGAAGACGG	62	75	NM_001277854.1
*GPX*	F: GGCTCGGTGTCGTTAGTTGT R: GCCCAAACTGATTGCATGGG	60	139	NM_001163245.1
*HMOX1*	F: ACACCCGCTATTTGGGAGAC R: GAACTTGGTGGCGTTGGAGA	62	134	NM_205344.1
*NQO1*	F: GAGCGAAGTTCAGCCCAGT R: ATGGCGTGGTTGAAAGAGGT	60.5	150	NM_001277619.1
*GST*	F: GCCTGACTTCAGTCCTTGGT R: CCACCGAATTGACTCCATCT	60	138	NM_001001776.1
*GSR*	F: GTGGATCCCCACAACCATGT R: CAGACATCACCGATGGCGTA	62	80	XM_015276627.1
*PRDX1*	F: CTGCTGGAGTGCGGATTGT R: GCTGTGGCAGTAAAATCAGGG	61	105	NM_001271932.1
*TXN*	F:ACGGAAAGAAGGTGCAGGAAT R: GATCCAGACATGCTCCGATGT	60	110	NM_205453.1

F—Forward; R—Reverse; *GAPDH*—glyceraldehyde 3-phosphate dehydrogenase; *ACTB*—actin beta; *Nrf2*—nuclear factor; erythroid 2-like 2; *KEAP1*—kelch-like ECH-associated protein 1; *CAT*—catalase; *SOD1*—superoxide dismutase 1; *XOR* —xanthine oxidoreductase; *GPX 2, 7*—glutathione peroxidase 2, 7; *HMOX1*—heme oxygenase 1; *NQO1*—NAD(P)H quinone dehydrogenase 1; *GST*—glutathione S-transferase; *GSR*—glutathione-disulfide reductase; *PRDX1*—peroxiredoxin-1; *TXN*—thioredoxin.

**Table 2:** Overall broiler growth performance responses.

	Overall BWG (g)	Overall FI (g)	Overall FCR (g FI/g BWG)
Diet type ^1^			
L	2411.7 ^A^	4114.9 ^A^	1.71 ^B^
H	2692.1 ^B^	4194.3 ^B^	1.56 ^A^
PFA addition ^2^			
No	2523.1 ^X^	4138.2	1.65 ^Y^
Yes	2580.7 ^Y^	4171.1	1.62 ^X^
Treatments (Interactions)			
L−	2353.3 ^a^	4086.3	1.74 ^c^
L+	2470.1 ^b^	4143.6	1.68 ^b^
H−	2692.8 ^c^	4190.1	1.56 ^a^
H+	2691.3 ^c^	4198.6	1.56 ^a^
SEM ^4^	16.78	38.20	0.013
*P*_D_^3^	<0.001	0.046	<0.001
*P*_P_^3^	0.002	0.395	0.043
*P*_D×P_^3^	0.001	0.528	0.024

^1^ Diet type: L (i.e., 95% of recommended ME and CP specs) and H (i.e., 100% of recommended ME and CP specs). ^2^ Phytogenic feed additive (PFA) supplementation (No = 0 mg/kg diet and Yes = 150 mg/kg diet). ^3^ Means within the same column with different superscripts per diet type (A, B), PFA addition (X,Y) and their interactions (a, b, c) differ significantly (*P* < 0.05). ^4^ Pooled standard error of means.

**Table 3:** Relative expression of the *Nrf2*/ARE pathway genes in the duodenal mucosa of 42-day-old broilers.

Item	Type of Diet ^2^		PFA Supplementation ^3^			*p*-Values ^4^		
Duodenum	L	H	No	Yes	SEM ^5^	Diet (D)	PFA (P)	D × P
Genes ^1^								
Nrf2	1.01	1.29	1.17	1.13	0.222	0.224	0.836	0.794
KEAP1	1.08	1.05	0.89 ^X^	1.25 ^Y^	0.096	0.753	0.001	0.125
CAT	2.22	2.15	1.78 ^X^	2.59 ^Y^	0.366	0.842	0.035	0.491
SOD1	1.02	1.14	0.92 ^X^	1.24 ^Y^	0.131	0.385	0.019	0.063
XOR	1.16	1.04	0.97	1.23	0.184	0.513	0.171	0.475
GPX2	1.69 ^B^	1.03 ^A^	1.06	1.66	0.313	0.043	0.063	0.170
GPX7	1.33	1.23	1.49	1.07	0.367	0.791	0.262	0.862
HMOX1	0.94 ^A^	1.19 ^B^	0.87 ^X^	1.26 ^Y^	0.101	0.017	0.001	0.017
NQO1	1.11	1.01	0.86 ^X^	1.25 ^Y^	0.109	0.368	0.001	0.183
GST	1.48	1.15	1.03	1.60	0.327	0.271	0.071	0.934
GSR	1.10	1.18	0.93 ^X^	1.34 ^Y^	0.194	0.664	0.041	0.062
PRDX1	1.77	1.62	1.38 ^X^	2.00 ^Y^	0.249	0.557	0.019	0.794
TXN	0.92 ^B^	1.26 ^A^	0.94 ^X^	1.24 ^Y^	0.136	0.019	0.035	0.120

^1^ Relative expression ratios of target genes was calculated according to [25] adapted for the multi-reference genes normalization procedure according to [26] using glyceraldehyde 3-phosphate dehydrogenase (*GAPDH*) and actin beta (*ACTB*) as reference genes. ^2^ Diet type: L (i.e., 95% of recommended ME and CP specs) and H (i.e., 100% of recommended ME and CP specs). Data shown per diet type represent treatment means from *n* = 18 broilers (e.g., for L diet, 9 from treatment L− and 9 from treatment L+). ^3^ Phytogenic feed additive (PFA) supplementation (No = 0 mg/kg diet and Yes = 150 mg/kg diet). Data shown for PFA represent means from *n* = 18 broilers (e.g., for No PFA supplementation, 9 from treatment L and 9 from treatment H). ^4^ Means within the same row with different superscripts per diet type (A, B) and PFA (X, Y) differ significantly (*P* < 0.05).^5^ Pooled standard error of means.

**Table 4:** Relative expression of the *Nrf2*/ARE pathway genes in the jejunal mucosa of 42-day-old broilers.

Item	Type of Diet ^2^		PFA Supplementation ^3^			*p*-Values ^4^		
Jejunum	L	H	No	Yes	SEM ^5^	Diet (D)	PFA (P)	D × P
Genes ^1^								
*Nrf2*	1.28	1.20	1.26	1.22	0.330	0.849	0.829	0.743
*Keap1*	1.09	1.07	1.07	1.09	0.109	0.926	0.886	0.430
*CAT*	1.17	1.00	1.08	1.09	0.126	0.180	0.951	0.719
*SOD1*	1.10	1.06	1.03	1.13	0.137	0.734	0.480	0.066
*XOR*	1.15	1.05	1.08	1.12	0.158	0.492	0.831	0.953
*GPX2*	1.46 ^B^	0.87 ^A^	1.00	1.33	0.138	0.005	0.108	0.294
*GPX7*	1.13	1.10	1.12	1.11	0.175	0.850	0.960	0.930
*HMOX1*	0.96	1.22	1.01	1.16	0.095	0.064	0.285	0.375
*NQO1*	1.04	1.08	1.09	1.03	0.088	0.766	0.628	0.793
*GST*	0.91	1.34	1.00	1.24	0.148	0.050	0.260	0.245
*GSR*	0.95	1.12	1.05	1.01	0.110	0.130	0.749	0.206
*PRDX1*	2.39 ^B^	1.63 ^A^	1.99	2.01	0.225	0.002	0.908	0.654
*TXN*	1.06	1.09	1.11	1.04	0.136	0.818	0.637	0.824

^1^ Relative expression ratios of target genes was calculated according to [25] adapted for the multi-reference genes normalization procedure according to [26] using glyceraldehyde 3-phosphate dehydrogenase (GAPDH) and actin beta (ACTB) as reference genes. ^2^ Diet type: L (i.e., 95% of recommended ME and CP specs) and H (i.e., 100% of recommended ME and CP specs). Data shown per diet type represent treatment means from *n* = 18 broilers (e.g., for L diet, 9 from treatment L− and 9 from treatment L+). ^3^ Phytogenic feed additive (PFA) supplementation (No = 0 mg/kg diet and Yes = 150 mg/kg diet). Data shown for PFA represent means from *n* = 18 broilers (e.g., for No PFA supplementation, 9 from treatment L and 9 from treatment H).^4^ Means within the same row with different superscripts per diet type (A, B) differ significantly (*P* < 0.05). ^5^ Pooled standard error of means.

**Table 5:** Relative expression of the *Nrf2*/ARE pathway genes in the ileal mucosa of 42-day-old broilers.

Item	Type of Diet ^2^		PFA Supplementation ^3^			*p*-Values ^4^		
Ileum	L	H	No	Yes	SEM ^5^	Diet (D)	PFA (P)	D × P
Genes ^1^								
*Nrf2*	1.33	1.05	1.35	1.03	0.226	0.220	0.166	0.114
*Keap1*	1.11	1.03	1.02	1.12	0.126	0.553	0.438	0.433
*CAT*	1.26 ^B^	0.91 ^A^	1.00	1.17	0.120	0.006	0.149	0.151
*SOD1*	1.11	1.00	1.06	1.05	0.113	0.336	0.903	0.981
*XOR*	1.05	1.08	1.04	1.08	0.128	0.836	0.757	0.724
*GPX2*	1.45	1.06	0.81 ^X^	1.70 ^Y^	0.251	0.657	<0.001	0.007
*GPX7*	1.10	1.21	1.23	1.08	0.224	0.633	0.507	0.779
*HMOX1*	0.96	1.20	0.97	1.19	0.134	0.096	0.115	0.024
*NQO1*	1.18	0.95	1.05	1.08	0.119	0.058	0.845	0.184
*GST*	1.08	1.42	0.98	1.51	0.347	0.477	0.629	0.333
*GSR*	1.22	0.94	1.01	1.15	0.153	0.078	0.375	0.994
*PRDX1*	1.65	2.06	1.57	2.14	0.289	0.163	0.060	0.125
*TXN*	1.03	1.13	1.08	1.08	0.155	0.528	0.994	0.599

^1^ Relative expression ratios of target genes were calculated according to [25] adapted for the multi-reference genes normalization procedure according to [26] using glyceraldehyde 3-phosphate dehydrogenase (GAPDH) and actin beta (ACTB) as reference genes. ^2^ Diet type: L (i.e., 95% of recommended ME and CP specs) and H (i.e., 100% of recommended ME and CP specs). Data shown per diet type represent treatment means from *n* = 18 broilers (e.g., for L diet, 9 from treatment L− and 9 from treatment L+). ^3^ Phytogenic feed additive (PFA) supplementation (No = 0 mg/kg diet and Yes = 150 mg/kg diet). Data shown for PFA represent means from *n* = 18 broilers (e.g., for No PFA supplementation, 9 from treatment L and 9 from treatment H). ^4^ Means within the same row with different superscripts per diet type (A, B) and PFA (X, Y) differ significantly (*P* < 0.05). ^5^ Pooled standard error of means.

**Table 6:** Relative expression of the *Nrf2*/ARE pathway genes in cecal mucosa of 42-day-old broilers.

Item	Type of Diet ^2^		PFA Supplementation ^3^			*p*-Values ^4^		
Ceca	L	H	No	Yes	SEM ^5^	Diet (D)	PFA (P)	D × P
Genes ^1^								
*Nrf2*	1.16	1.14	1.18	1.13	0.204	0.910	0.787	0.718
*Keap1*	1.31 ^B^	0.93 ^A^	1.01	1.24	0.147	0.014	0.133	0.077
*CAT*	1.05	1.24	0.91	1.38	0.255	0.454	0.077	0.202
*SOD1*	1.13	1.11	1.04	1.20	0.172	0.926	0.352	0.135
*XOR*	1.21	1.07	1.00	1.28	0.262	0.402	0.393	0.359
*GPX2*	1.40 ^B^	0.86 ^A^	1.02	1.24	0.166	0.003	0.198	0.102
*GPX7*	1.39 ^B^	0.94 ^A^	1.08	1.25	0.201	0.032	0.406	0.144
*HMOX1*	1.22	1.04	1.12	1.15	0.152	0.247	0.865	0.991
*NQO1*	1.11	1.09	1.17	1.03	0.148	0.911	0.359	0.454
*GST*	1.34	0.99	0.98 ^X^	1.35 ^Y^	0.175	0.058	0.041	0.461
*GSR*	1.10	1.23	1.24	1.10	0.207	0.522	0.505	0.191
*PRDX1*	1.94 ^B^	1.29 ^A^	1.44	1.79	0.221	0.006	0.127	0.962
*TXN*	1.21	1.05	0.99	1.27	0.199	0.445	0.167	0.560

^1^ Relative expression ratios of target genes were calculated according to [25] adapted for the multi-reference genes normalization procedure according to [26] using glyceraldehyde 3-phosphate dehydrogenase (GAPDH) and actin beta (ACTB) as reference genes. ^2^ Diet type: L (i.e., 95% of recommended ME and CP specs) and H (i.e., 100% of recommended ME and CP specs). Data shown per diet type represent treatment means from *n* = 18 broilers (e.g., for L diet, 9 from treatment L− and 9 from treatment L+). ^3^ Phytogenic feed additive (PFA) supplementation (No = 0 mg/kg diet and Yes = 150 mg/kg diet). Data shown for PFA represent means from *n* = 18 broilers (e.g., for No PFA supplementation, 9 from treatment L and 9 from treatment H). ^4^ Means within the same row with different superscripts per diet type (A, B) and PFA (X, Y) differ significantly (*P* < 0.05). ^5^ Pooled standard error of means.

**Table 7:** Total antioxidant capacity (TAC) along the intestinal mucosa of 42-day-old broilers.

TAC ^1^ (mmol TE/g Tissue)	Type of Diet ^2^		PFA Supplementation ^3^		SEM ^4^	*p*-Values ^5^		
Item	L	H	No	Yes		Diet (D)	PFA (P)	D × P
Duodenum	46.46	46.53	44.38	48.60	2.279	0.976	0.073	0.024
Jejunum	39.22 ^A^	52.38 ^B^	38.32 ^X^	53.29 ^Y^	3.748	0.001	<0.001	0.250
Ileum	32.53	34.31	29.73 ^X^	37.11 ^Y^	3.316	0.594	0.033	0.007
Ceca	33.94	39.03	32.49 ^X^	40.48 ^Y^	3.564	0.163	0.032	0.300

TE = trolox equivalents. ^1^ Data represent treatment means from *n* = 9 replicate floor pens per treatment (L, L+, H, H+). ^2^ Diet type: L (i.e., 95% of recommended ME and CP specs) and H (i.e., 100% of recommended ME and CP specs). Data shown per diet type represent treatment means from *n* = 18 broilers (e.g., for diet type L, 9 from treatment L− and 9 from treatment L+). ^3^ Phytogenic feed additive (PFA) supplementation (No = 0 mg/kg diet and Yes = 150 mg/kg diet). Data shown for PFA represent means from *n* = 18 broilers (e.g., for No PFA supplementation, 9 from treatment L and 9 from treatment H). ^4^ Pooled standard error of means. ^5^ Means within the same row with different superscripts per diet type (A, B) and phytogenic feed additives (X, Y) differ significantly (*P* < 0.05).

## Data Availability

The data analyzed during the current study are available from the corresponding author on reasonable request.
